# Regulation of Inflammatory Responses by Pulsed Electromagnetic Fields

**DOI:** 10.3390/bioengineering12050474

**Published:** 2025-04-30

**Authors:** Amr Kaadan, Simona Salati, Ruggero Cadossi, Roy Aaron

**Affiliations:** 1 Department of Orthopaedic Surgery, Warren Alpert Medical School of Brown University, Providence, RI 02903, USA; amro_kaadan@alumni.brown.edu; 2 Medical Division, Igea S.p.A., 41012 Carpi, Italy; s.salati@igeamedical.com (S.S.); r.cadossi@igeamedical.com (R.C.)

**Keywords:** pulsed electromagnetic fields, inflammation, wound healing, diabetic wound healing, venous ulcers, angiogenesis, cartilage

## Abstract

Pulsed Electromagnetic Field (PEMF) therapy has been shown to have substantial suppressive effects on inflammation and is a promising treatment for the modulation of inflammation. Several in vitro and in vivo studies have shown that PEMFs profoundly suppress inflammatory pathways, such as the NF-κB and MAPK signaling pathways, by lowering cytokine levels and improving extracellular matrix synthesis. This review describes studies, ranging from in vitro to clinical, that investigate the lesser-known roles of PEMF in the modulation of inflammation in soft tissue wound, cartilage, and joint healing, alongside angiogenesis. Mechanistically, PEMFs act via adenosine receptors, specifically A_2A_, which play a key role in inflammation modulation and tissue repair. In some clinical trials, PEMF has yielded short-term symptom relief and functional improvements in early-stage osteoarthritis patients, arthroscopy patients, and anterior cruciate ligament reconstruction patients.

## 1. Introduction

Intercellular communication or signaling in biological systems is essential to permit the organism to function in an integrated fashion. While there are many forms of cell communication, two types of signaling are especially prominent: chemical signaling (including gap junction signaling, adhesive intercellular interactions, endocrine, paracrine, and autocrine signaling) and biophysical communication through mechanical and electrical signals. In animal systems, electrical signaling is robust and occurs in organ systems, notably neurological and cardiac systems, and at the cellular level in a variety of stem cells. In the musculoskeletal system, mechanical signaling and electrical signaling are interwoven as mechanical strain induces the flow of fluid and ions, producing endogenous electrical currents that act as signaling events for the synthesis of cytokines or structural proteins, often culminating in extracellular matrix remodeling. The external application of electrical and electromagnetic fields signals a variety of biological events, notably in the skeleton and Pulsed Electromagnetic Field (PEMF) is widely used clinically for bone repair. Chemical and physical signaling often act in concert especially when transducing external physical stimuli. In addition to the well-known use of PEMF for bone repair, however, PEMFs have a plethora of effects on inflammation and on soft tissue wound repair. The purpose of this review to present some of these lesser-known effects of PEMF, describing what is known of their mechanisms, in the hopes that the range of therapeutic effects of PEMF will be considered to control articular and other sources of inflammation and for the enhancement of soft tissue wound repair.

## 2. Effects of PEMF on Inflammation

Pulsed Electromagnetic Field (PEMF) therapy has gained significant attention as a non-invasive, cost-effective treatment to manage fracture nonunion. Additionally, preclinical and some clinical data have suggested that PEMFs may also have an anti-inflammatory effect and may be useful in stimulating the healing of soft tissue wounds. The cell membrane has been identified as the target and site of interaction of PEMF stimulation. Through the cell membrane, the physical signal is transduced by a series of intracellular chemical second messengers that result in the biological response. The specific mechanism of interaction between PEMFs and the cell membrane was described in 2002 by Varani et al., identifying the adenosine receptors (ARs) as one of the possible targets of PEMF stimulation [1]. ARs play a pivotal role in the regulation of inflammatory processes, with both pro- and anti-inflammatory effects [2]. Adenosine is a purine endogenous nucleoside with several functions involved in pain, inflammation, and neurodegenerative diseases. Extracellular adenosine levels are usually low under a physiological condition; however, during inflammation or in hypoxic/ischemic conditions, the extracellular concentration of adenosine increases [3]. Adenosine mediates its effects through its interaction with four types of G-protein coupled receptors: A_1_, A_2A_, A_2B_, and A_3_ARs. A_1_ and A_3_ ARs are coupled to G_i_ proteins and inhibit adenylate cyclase (AC), reducing intracellular cAMP levels; A_2A_ and A_2B_ ARs are coupled to G_s_ proteins, and their activation leads to an increase in intracellular cAMP [4]. AR modulation is strongly implicated in the regulation of inflammatory processes, suggesting their involvement in different pathologies resulting from inflammation [2].

Varani et al. demonstrated that PEMF exposure induces a significant increase in A_2A_ and A_3_ AR density on the cell membrane of human neutrophils [1]. Notably, A_1_ and A_2B_ receptors were not influenced by the same exposure conditions. Moreover, in the presence of the specific A_2A_ receptor agonist, PEMF exposure was able to synergize with the agonist and induce a significant increase in intracellular cAMP levels. On the contrary, the presence of the specific A_2A_ receptor antagonist blocked the effects of both the agonist and PEMF stimulation, suggesting that PEMFs specifically act through the activation of A_2A_ adenosine receptors with a pharmacologic-like mechanism. These results have been subsequently confirmed in different cell types, chondrocytes [5], synoviocytes [5,6,7,8] and osteoblasts [9], (discussed in more detail in Section 3.3 below), and they are the mechanism of action underlying the anti-inflammatory and protective effects of PEMFs in inflammatory and degenerative tissue diseases. The agonist activity of PEMFs for the A_2A_ and the A_3_ adenosine receptors leads to the inhibition of the NF-kB signaling pathway, which is a pivotal regulator of the inflammatory response [7], regulating the expression of several pro-inflammatory genes, which encode for inflammatory cytokines and chemokines.

The strong anti-inflammatory action of PEMFs also suggests potential new clinical applications for PEMFs in treating a range of diseases characterized by chronic low-grade inflammation, which is associated with cellular responses including senescence. Several studies have linked senescence with inflammation [8]: senescent cells release different factors known as the senescence-associated secretory phenotype (SAPS) factors, such as pro-inflammatory mediators and metalloproteinases into the surrounding microenvironment. These molecules further promote the propagation of a chronic low-grade inflammatory state, which, in turn, drives the spread of additional senescence. In this context, stimulation with PEMFs could play a role in breaking the vicious cycle underlying inflammaging [10]. PEMF stimulation has also been suggested as an innovative treatment to promote tissue regeneration [11]. The inflammatory response following tissue damage is crucial in determining the success of the healing process, including the extent of scarring and the restoration of physiological tissue functions. When poor regulation of the inflammatory response permits acute phase inflammation to evolve into chronic inflammation, tissue, regeneration is compromised: PEMF treatment, by modulating the inflammatory processes, might enhance the functional recovery of injured tissues.

## 3. Effects of PEMF on Inflammation in Soft Tissue Wound Healing

Wounds can be categorized into acute and chronic based on their healing time. Wound healing is complex, involving various cells and culminating in the restoration of the extracellular matrix (ECM) [12]. Wound healing begins immediately following injury and can be divided into three phases. The first is the inflammatory phase which begins with coagulation, hemostasis, and cytokine signaling. The coagulation cascade promotes the platelet plug, which then leads to platelet aggregation accompanied by the release of numerous chemokines, notably, platelet-derived growth factor (PDGF) and transforming growth factor beta (TGF-β). These mediators provide chemotactic signals that draw neutrophils to the wound site [13]. During this phase, macrophages play a critical role in establishing homeostasis and modulating inflammation to prevent it from becoming pathological. The proliferative phase follows the inflammatory phase, normally lasts up to 21 days, and is characterized by ECM formation, angiogenesis, and epithelization. In this phase, platelets degranulate due to PDGF’s promotion of collagen and proteoglycan formation [13], while fibroblasts transform into wound-contracting myofibroblasts, also from PDGF [13]. In this phase, keratinocyte-derived growth factor (KGF) stimulates epithelialization from keratinocytes. In addition, vascular endothelial growth factor (VEGF) and basic fibroblast growth factor (bFGF) are produced, both of which promote the angiogenesis of blood vessels. The final phase of normal wound healing is the maturation phase, which can last up to a year following the initial injury. This phase is characterized by replacing type III collagen with type I collagen. The successful completion of the maturation phase depends on adequate tissue oxygenation around the wound’s edges [13]. Many issues may arise that can negatively affect wound healing such as infection, trauma, and preexisting medical conditions. This section will describe three wound healing conditions, diabetic wounds (DWs), venous ulcers (VUs), and vascular damage, which can be improved by exposure to PEMF.

### 3.1. Diabetic Wound Healing

**Figure 1:**
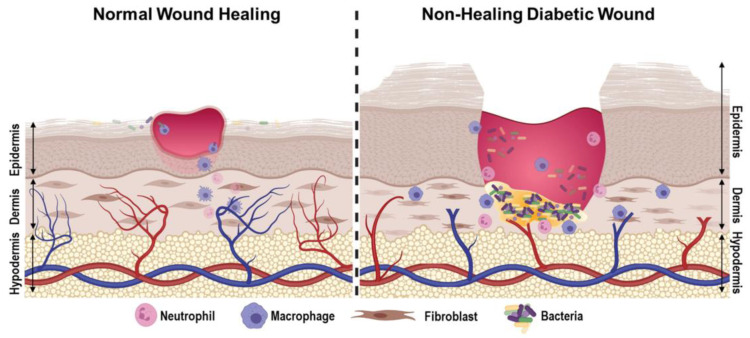
A comparison between normal (**left**) and DW healing (**right**). In the DW healing diagram, impaired angiogenesis, bacterial colonization, and an inadequate inflammatory response are present, showcasing the complex pathophysiology of these wounds. The figure is from Burgess et al. [14].

DW healing differs from standard wound healing because of the pathophysiology presented by diabetes [14]. In DW healing, the inflammatory response is inadequate, angiogenesis is deregulated, reactive oxygen species levels are elevated, the proliferation of keratinocytes and fibroblasts is disrupted, there is decreased collagen deposition, and the presence of difficult-to-treat bacterial colonization creates a unique challenge to heal DWs [14]. Figure 1 provides a comparison between normal wound healing and DW healing.

A systematic review reported on five trials that investigated PEMF effects on DW healing with the main outcome being wound closure percentage [15]. In four of those trials, all PEMF groups showed improved wound healing in comparison to controls, demonstrating the enhancement of DW healing by exposure to PEMF [15]. One of the examined trials measured cell proliferation and vascularity and demonstrated improved cell proliferation (Experimental: 31.5 ± 5 vs. Control: 7.52 ± 8 per high-power field in db/db mice) and vascularity (E: 28 ± 4 vs. C: 17 ± 4 cells per high power field) PEMF treatment. The PEMF parameters were 4.5 ms pulses at 15 Hz for 8 h/daily. Another trial investigated collagen deposition and found significantly greater type 1 collagen amount and deposition (*p* = 0.013) and myofibroblast population numbers following PEMF treatment (*p* = 0.024) compared to a control group with no PEMF activation [15].

Choi et al. examined the effects of 2 and 10 mT PEMF intensities at 25 Hz on wound healing in diabetic rats. The rats were randomly allocated into sham and active PEMF groups, and the experiment was conducted over a period of 21 days [16]. The measured outcomes consisted of maximum load, maximum stress, energy absorption capacity, Young’s modulus and wound tissue thickness. PEMF was shown to promote the recovery of structural properties of diabetic rat wounds by increasing overall wound tissue thickness through the promotion of cell proliferation and collagen deposition [16]. The higher intensity 10 mT PEMF treatment provided significantly greater wound thickness compared to the sham group (*p* = 0.007). However, the lower intensity 2 mT PEMF treatment may potentially be more effective in enhancing wound closure in the early stages of wound healing, as the wounds were significantly smaller than those of the sham group (*p* = 0.02). Interestingly, PEMF may not be as effective in the remodeling phase after re-epithelization occurs as neither PEMF intensity was able to improve mechanical properties, including Young’s modulus. PEMF showed great efficacy in enhancing ECM formation in the dermis and sub-dermis. It seemed to have little clinically relevant effect on epithelial closure [16].

Goudarzi et al. examined the effects of 8 mT PEMF intensity at 20 Hz applied for 1 h/day over 10 days on diabetic rat wound healing [17]. Diabetic rats were divided into two groups of seven: a PEMF-treated group and a sham group. Additionally, a diabetes control group of nondiabetic rats was also divided into two groups of seven: a PEMF-treated group and a sham group. Wound healing was measured by the duration of healing, wound surface area, and tensile strength. Scar tensile strength was measured by incising a rectangular skin segment, then preparing the segment with saline solution, and immediately testing the specimen with a tensiometer.

The results showed that PEMF treatment significantly reduced wound healing time (*p* < 0.001) and improved scar tensile strength (*p* < 0.001) in diabetic rats (Table 1). In addition, the diabetic rats were then compared to the diabetes control groups, and the results showed that PEMF reduced wound healing time (*p* < 0.01) and improved scar tensile strength (*p* < 0.05) to the point where they were not statistically significant compared to the diabetes control group [17]. Table 1 summarizes data from Goudarzi et al. The means but no variances are supplied.

### 3.2. Venous Ulcer Healing

**Figure 2:**
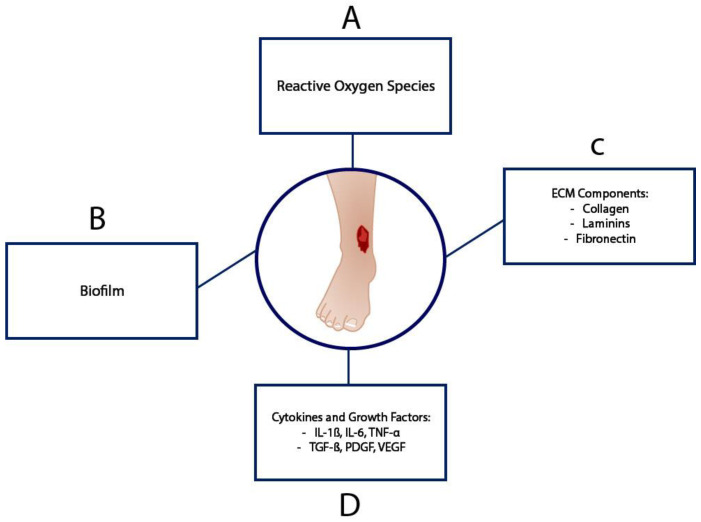
Major biochemical compounds that affect healing in the complex VU microenvironment. (**A**) Reactive oxygen species are generated due to iron overload. (**B**) Biofilm builds up as a result of wound site infection. (**C**) ECM components are dysregulated under inflammatory conditions. (**D**) Pro-inflammatory cytokines and growth factors are present. The figure is adapted from Coelho et al. [19].

VUs are the most common type of chronic lower extremity ulcers and are thought to primarily be a result of venous hypertension or obstruction [18]. VUs typically occur from a complex process secondary to venous hypertension followed by inflammation within venous circulation and venous structures. VUs typically present as irregular and shallow with well-defined borders and are commonly found over bony prominences [18]. Figure 2 presents a brief overview of the biochemical pathophysiology of VUs.

PEMF treatment may enhance VU healing according to a randomized controlled trial that investigated the combined effects of PEMF and plantar flexion resistance exercise on venous leg ulcers [20]. In this study, sixty patients were divided into three groups: PEMF with resistance exercise, PEMF with conservative treatment, and a control with conservative treatment only. The conservative treatment was plantar flexion resistance exercise. Significant improvements were observed in the VU surface area and volume in both experimental groups when compared to the control group (*p* < 0.05) [20]. Ieran et al. conducted a double-blind study that investigated the effects of PEMF on VUs in 44 patients for up to 90 days [21]. At the 90-day mark, the PEMF group had a significantly higher success rate compared to the control group (*p* < 0.02) and had a significantly higher success rate in the follow-up period. Within 1 year from the start of the stimulation, 8 out of 19 patients had healed among the controls, with an overall success rate of 42.1%, and 16 out of 18 patients in the active group had an overall success rate of 88.8%, significantly higher than the controls (*p* < 0.005), suggesting that PEMF’s therapeutic effects may last beyond the treatment period [21]. Stiller et al. conducted a randomized, double-blind, placebo-controlled multicenter study that investigated the clinical efficacy and safety of pulsed electromagnetic limb ulcer therapy (PELUT), a PEMF treatment, in the treatment of recalcitrant venous ulcers [22]. The 8-week study applied PELUT for 3 h/day as an adjunct treatment to wound dressing and assessed wound surface area, ulcer depth, and pain levels at three intervals. At the 8-week mark, the results showed that the active PEMF group had a 47.7% decrease in wound surface area vs. a 42.3% increase for the placebo group (*p* < 0.0002). In addition, wound depth (*p* < 0.04) and pain levels (*p* < 0.04) were both significantly decreased in the PELUT group in comparison to the placebo group. The authors concluded that PELUT is safe and effective as an adjunct therapy for recalcitrant venous leg ulcers [22].

### 3.3. Hypoxic and Vascular Damage

**Figure 3:**
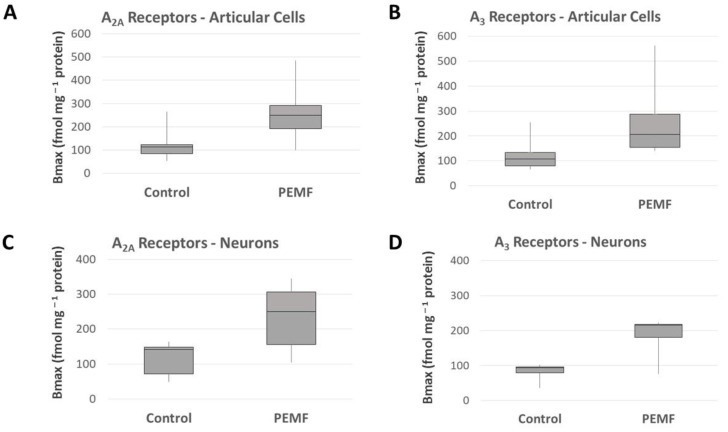
A_2A_ (**A**) and A_3_ (**B**) ARs density in human and bovine chondrocytes, synoviocytes and osteoblasts, box plot showing median receptor density are presented. A_2A_ (**C**) and A_3_ (**D**) ARs density in rat cortical neurons, PC12 cells, NGF-treated PC12 cells, rat cerebral cortex and U87-MG cells, box plot showing median receptor density are presented. Data from references [5,7,8,9].

The anti-inflammatory and tissue-preserving effects exerted by PEMFs through specific action on adenosine receptors show great therapeutic potential in controlling brain inflammation and providing neuroprotection following ischemia and hypoxic brain damage. In order to assess whether PEMF stimulation was able to act on neuronal cells by modulating ARs, Varani et al. analyzed rat cerebral cortex tissue samples, cerebral cortex membranes and cortical neurons through saturation binding experiments [23]. In intact rat cerebral cortex and cortical neurons, PEMF stimulation induced a time and dose-dependent transient increase in A_2A_ ARs. Later, Vincenzi et al. confirmed these results in PC12 rat adrenal pheochromocytoma and U87MG human glioblastoma cell lines (Figure 3C,D) [24]. Using A_2A_ and A_3_ AR selective agonists, the authors were able to show that PEMF stimulation enhances the effect of AR agonists on cAMP production, demonstrating that PEMFs act by augmenting the activity of endogenous adenosine. The results in Nerve Growth Factor (NGF)-treated PC12 and U87MG cells also confirmed that the PEMF stimulation of A_2A_ and A_3_ ARs led to significant reduction in NF-kB signaling activation.

This mechanism of action underlies the main effects of PEMF stimulation on neuronal cells described so far: (1) the inhibition of inflammation, (2) protection from apoptosis, and (3) the promotion of neuronal plasticity. In agreement with the findings on joint cells, PEMF exposure is also able to control the inflammatory response in cells of neuronal origin. Varani’s group showed that PEMF stimulation reduced the release of several pro-inflammatory cytokines (tumor necrosis factor–α, TNF-α, IL-1β, IL-6, and IL-8) in LPS-activated N9 microglial cells [25]. PEMFs have also been shown to decrease ROS generation, cell invasion, and phagocytosis in activated microglial cells [26], thus mitigating neuro-inflammation following ischemic damage.

The stimulation of A_2A_ ARs has also been reported to protect neurons from cell death triggered by neurotoxins [27]; thus, the agonist effect of PEMFs on A_2A_ ARs can be exploited to promote neuron survival and viability. Osera et al. reported the ability of PEMF exposure to enhance cellular defense against oxidative stress in the human neuroblastoma SH-SY5Y cell line [28]. In neuron-like cells, PEMF exposure significantly reduced apoptosis, partially restored hypoxia inducible factor-1α (HIF-1α) activation to normal conditions and inhibited ROS production [25]. In hypoxic PC12 cells, PEMFs activated the p38 kinase cascade, increasing chaperone heat-shock proteins of 70 kDa (HSP70) and cAMP response element-binding protein (CREB) phosphorylation, leading to elevated brain-derived neurotrophic factor (BDNF) and Bcl-2 expression, thus promoting neuronal survival [29]. Moreover, PEMF exposure also increases the neurotrophic and protective action of astrocytes in response to hypoxic damage: PEMFs have been shown to induce Vascular Endothelial Growth Factor (VEGF) release in the human astrocyte cell line 1321N1, whose conditioned medium prevented viability loss in oxygen-deprived SH-SY5Y neuron-like cells [30]. These findings support the role of PEMF exposure in protecting neurons from hypoxic-induced cell death.

Of additional interest is the capacity of PEMF stimulation to promote neuronal differentiation and neurite outgrowth. Zhang et al. found PEMFs (1.36 mT, 50 Hz) increased neurite length in PC12 cells [31] through MEK-ERK1/2 activation [32]. More recently, Fontana et al. reported that PEMF exposure promotes neurite outgrowth in neuroblastoma F11 cells, suggesting neurogenesis promotion and acceleration after PEMF exposure [33]. Similarly, PEMFs enhanced neurite outgrowth in MN9D dopaminergic neurons: the authors also reported increased intracellular cAMP levels within 3 to 5 h from treatment, suggesting cAMP formation as a potential mechanism of action [34]. Interestingly, the A_2A_ receptor is coupled with a G_S_ protein, which activates adenylate cyclase, inducing a significant increase in the cAMP intracellular level. In this view, PEMF stimulation could contribute to brain tissue remodeling after cerebral ischemia.

These in vitro results provide the rationale supporting the preliminary observations made by Grant et al. in a rabbit model of cerebral ischemia, where PEMF exposure (2.8 mT, 75 Hz, 4 h) reduced the ischemic area by 65% and 69% as shown via MRI and histology, respectively [35]. Similar reductions in necrotic areas were observed in rodent myocardial infarct (MI) models [36,37]. PEMF treatment promoted the angiogenesis of the infarct border zone and improved cardiac function in MI mice [37]. In skin-free flap experiments, which can be regarded as models of terminal vascularization, devoid of collateral circulation, PEMF exposure stimulated angiogenesis, thus promoting tissue survival [38].

Recent studies confirmed that PEMF reduces ischemic lesion volume and modulates inflammatory parameters in a murine stroke model, showing anti-inflammatory and anti-apoptotic effects [39]. Additionally, PEMFs decreased IL-1β and MMP-9 levels while increasing pro-survival molecules via the TrkB/Akt/Bad pathway [40]. Despite substantial preclinical evidence, clinical studies on stroke patients are limited. Cichon et al. showed that PEMF treatment (7 mT, 40 Hz, 15 min/day for 4 weeks) improved patient clinical parameters, particularly cognitive and psychosomatic functions [41] and increased antioxidant enzyme activity, thus reducing the oxidative stress level [42]. PEMFs also promoted neuroplasticity by enhancing BDNF and VEGF levels, consistent with preclinical findings [43]. On the contrary, the same authors also reported higher expression levels of IL-1β [44] and pro-apoptotic genes [45] in PEMFs-treated patients, possibly indicating the activation of neuroprotective and plasticity pathways; however, the underlying mechanism requires further clarification. It is also worth noting that Cichon’s studies applied PEMF stimulation to the pelvic area rather than the brain, and the multiple measurements reported were conducted on the same group of patients, limiting their generalizability. Based on the anti-inflammatory and neuroprotective effects shown in pre-clinical studies, Capone et al. conducted an exploratory trial on six acute ischemic stroke patients, exposing them to PEMFs within 48 h from stroke onset for five consecutive days. The treatment was well tolerated, with no severe adverse events. Magnetic Resonance Imaging (MRI) results showed lesion volume reduction in one patient treated for 45 min/day and in all patients treated for 120 min/day [46]. Dosimetric analysis revealed a correlation between magnetic field intensity (≥1 mT) and lesion reduction, suggesting a possible dose–response effect [47]. These preliminary findings opened the way to a randomized, placebo-controlled trial (NCT02767778) to evaluate PEMFs’ ability to promote recovery in acute ischemic stroke patients, whose results have been recently published [48]. The study enrolled 34 ischemic stroke patients: 14 in the active group and 20 in the placebo group. The results showed that the brain lesion volume identified by MRI was significantly reduced at 45 days compared to baseline in PEMF-treated patients only (*p* = 0.02), whereas no statistically significant reduction was observed in the placebo group. Among study participants receiving reperfusion therapy, PEMF-treated patients achieved 50% reduction in lesion volume compared to 22.7% in the placebo group (*p* = 0.046). Clinical scores also revealed faster and larger improvements in the active treatment group. By day 90, an excellent outcome—defined as an mRS score of 0 to 1—was achieved in 90.9% of patients in the active group compared to 66.7% in the placebo group. No patients showed serious adverse events during or at the end of treatment, nor possible late effects during follow-up, confirming PEMF treatment safety and tolerability in stroke patients. These results suggest that PEMF can be applied to stroke patients as a neuroprotective treatment, by tackling the inflammatory processes following the ischemic insult, to prevent the enlargement of the neuronal damage to the penumbra surrounding the infarct core, promoting the reduction in the brain lesion volume, ultimately favoring patients’ clinical recovery.

## 4. Effects of PEMF on Inflammation in Cartilage and Joints

Osteoarthritis (OA) and its accompanying cartilage degeneration represent considerable clinical challenges, largely attributable to the poor regenerative capacity of cartilage. Inflammation in OA synovium is physiologically complex, encompassing multiple cell and tissue types around and within the joints [49]. The inflammatory reaction impacts a number of tissues such as articular cartilage and subchondral bone [49]. Pro-inflammatory cytokines IL-1β and TNF-α induce the activation of NF-κB and MAPK signaling pathways, resulting in the increased expression of matrix metalloproteinases (MMPs) responsible for the degradation of extracellular matrix (ECM) constituents, namely type II collagen and proteoglycans in articular cartilage [50,51,52,53,54]. Type II collagen, along with many other ECM fragments, acts as damage-associated molecular patterns (DAMPs) that enhance the catabolic reactions observed in chondrocytes [55,56].

Numerous in vitro and clinical studies have investigated the role of PEMF in cartilage repair and joint healing. PEMF has been shown to be a promising non-invasive intervention in promoting cartilage repair by reducing inflammation and enhancing chondrogenesis.

PEMF stimulation, through the inhibition of the NF-kB pathway, decreases the release of IL-6 and IL-8 in human chondrocytes [9] and the synthesis of prostaglandin E2 (PGE2) and cyclooxygenase type 2 (COX-2) in bovine synoviocytes [8]. In human synovial fibroblasts from osteoarthrosis patients, PEMF stimulation lowers the synthesis of inflammatory mediators such as PGE2, IL-6 and IL-8 while stimulating the release of the anti-inflammatory cytokine IL-10 [7] thus potentially reducing inflammation and cartilage degradation. More recently, Fontana et al. showed that PEMF exposure was able to decrease the release of TNF-α and IL-8 from M1-differentaited macrophages while increasing the release of IL-10 in THP-1 undifferentiated macrophages [33].

In vivo, in the synovial fluid of sheep treated with PEMF stimulation as an adjunct to autologous osteochondral grafts, significantly lower levels of pro-inflammatory cytokines, IL-1β and TNF-α, and a higher concentration of TGF-β1 were measured in stimulated animals compared to controls [57]. Moreover, PEMF stimulation also favored graft integration and prevented graft reabsorption after autologous osteochondral grafts. In fact, through their action on ARs, PEMFs have also been shown to exert pro-anabolic activity: in vitro PEMF stimulation mediated a significant increase in chondrocyte and osteoblast proliferation [9]. Interestingly, several reports have shown that PEMFs significantly increased chondrocyte proliferation and the synthesis of specific cartilage components including proteoglycans and collagen type II [58,59], showing a strong anabolic effect. Moreover, PEMFs exert a protective effect against the catabolic activity of pro-inflammatory cytokines: in both human and bovine articular cartilage explants, PEMF stimulation was able to counteract the catabolic activity of IL-1β and increase PG synthesis [58,59]. Additionally, PEMFs have been shown to counteract the IL-1β-induced inhibition of chondrogenesis in mesenchymal stem cells, suggesting that electromagnetic stimulation could be successfully applied to reduce the catabolic effects of pro-inflammatory cytokines within the cartilage environment in OA patients [60].

Altogether, these results constitute the rationale for PEMF application in inflammatory-based degenerative diseases, showing that PEMF stimulation increases anabolic activity while preventing the catabolic effects of inflammation through AR mediation.

**Figure 4:**
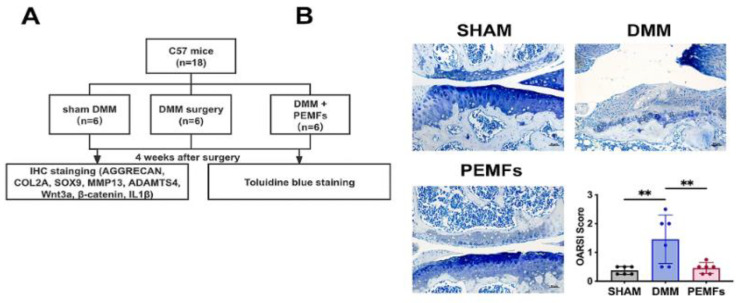
PEMF exerts cartilage protective effects in an OA mouse model and inhibits Wnt-βcatenin signaling. (**A**) Experimental group schematic. (**B**) Representative toluidine blue-stained joint sections from mice. Sham: Demonstrates normal articular and meniscal cartilage. DMM surgery: Demonstrates reduced toluidine blue staining in the articular cartilage. PEMF + DMM: Cartilage morphology and staining are grossly normal. OARSI scores show statistically significant differences between sham and DMM-only groups and between PEMF and DMM-only groups and are represented by **. Figure from Song et al. [61] and is cropped.

In in vitro studies, PEMF was able to downregulate pro-inflammatory cytokines such as IL-1β, IL-6, and IL-8 and promoted pathways involved in ECM synthesis. Song et al. have shown that PEMF is able to preserve chondrogenic differentiation under inflammatory knee OA conditions through the inhibition of NF-κB pathway activation in bone marrow mesenchymal stem cells (BMSCs) and the inhibition of the Wnt/β-catenin signaling pathway [61]. The destabilization of the medial meniscus surgery (DMM) was performed on the right knee of mice for OA induction. Applying PEMF at 75 Hz and 3 mT for 7 days showed increased chondrogenic differentiation by the enhanced expression of ECM markers, ACAN, COL2A, and SOX9, while observing a reduction in MMP3 and MMP13 [61]. Figure 4 describes the cartilage protection effects of PEMF in the mouse model.

Similarly, PEMF-treated adipose-derived MSCs (AMSCs) released exosomes that resisted inflammation in chondrocytes [62]. Exosomes are small, membrane-bound extracellular vesicles that contain proteins, lipids, and nucleic acids. Exosomes are released from various cell types and can play a crucial role in intracellular communication [63]. In this study, using IL-1β -induced chondrocytes, PEMF-exposed AMSC-derived exosomes substantially suppressed inflammation and ECM degradation compared to non-PEMF-exposed AMSC-derived exosomes as shown by the higher expression of transcripts and proteins of COL2A1, SOX9, and ACAN and lower expression of MMP13 and caspase-1 [62]. In addition, the PEMF stimulation of chondrocytes directly inhibited IL-1β-driven cartilage breakdown through the regulation of the MAPK/ERK pathway [64]. These findings were also shown in preclinical OA animal models treated with PEMF, which exhibited decreased joint inflammation and cartilage degradation. The PEMF treatment of OA guinea pig models demonstrated a noticeable alleviation of cartilage fibrillation and subchondral bone changes at a PEMF frequency of 75 Hz [65]. In OA rat models, early PEMF treatment was able to preserve cartilage integrity and minimize inflammatory damage [66].

In clinical studies, PEMF produced symptomatic and functional improvements in early-stage OA patients, but the long-term results were inconsistent. In a clinical review, Massari et al. highlighted PEMF’s ability to reduce inflammatory markers, such as PGE2, COX-2, and MMP-13, as previously discussed in this paper, highlighting its potential as a non-invasive anti-inflammatory therapy [67]. Gobbi et al. conducted a case series of 22 OA patients who underwent a 45-day PEMF treatment [68]. All patients presented with symptomatic early OA with grade 0–2 changes on the Kellgren–Lawrence classification during pretreatment evaluation. Patients were also evaluated at 1- and 2-year follow-ups using a visual analog scale for pain, International Knee Documentation Committee objective, Tegner, and Knee Injury and Osteoarthritis Outcome Scores (KOOSs). The results showed a significant improvement in all scores at the 1-year follow-up (*p* = 0.008), while the results deteriorated, but were still superior to pretreatment levels, at the 2-year follow-up (*p* = 0.02). The authors described how PEMF use in early symptomatic OA patients can lead to a significant improvement in OA symptoms, knee function, and activity at the 1-year follow-up [68]. Benazzo et al. conducted a randomized, prospective, double-blind study to evaluate the effects of PEMF in 69 patients undergoing arthroscopic anterior cruciate ligament (ACL) reconstruction [69]. Arthroscopic ACL reconstruction can generate an inflammatory reaction that may be harmful to articular cartilage [69]. This study evaluated the functional recovery of patients by the International Knee Documentation Committee (IKDC) form and non-steroidal anti-inflammatory drug (NSAID) use. All patients underwent the same ACL reconstruction method with use of the quadruple hamstrings semitendinosus and gracilis technique. The SF-36 Health Survey was used to follow up with patients at 30, 60, and 180 days, in addition to a 2-year follow-up interview. Patients were randomized to either an active PEMF (1.5 mT at 75 Hz) or a placebo group and were instructed to use the device for 4 h/day over a 60-day period. The results showed a significant decrease in SF-36 scores at 30 days in both groups (*p* < 0.0005). At 60 days, the mean SF-36 scores exceeded baseline scores by 3.2 points in the PEMF group, while scores were below baseline at −0.7 points in the placebo group. At 180 days, both groups experienced significant increases in SF-36 scores, with a 10.1 point increase over baseline in the PEMF group and a 7.2 point increase over baseline in the placebo group. Overall, the mean SF-36 survey results were significantly higher in the PEMF group compared to the placebo (*p* < 0.05) [69]. In addition, NSAID use was found to be less frequent in the PEMF group compared to controls (*p* < 0.05), joint swelling returned to normal faster in the PEMF group (*p* < 0.05). There were no statistically significant differences in the 2-year follow-up between the two groups. The authors concluded that PEMF use should be considered post-ACL reconstruction, particularly in professional athletes, to limit the inflammatory reaction within the joint, reduce recovery time, and ultimately preserve the joint [69].

Zorzi et al. conducted a randomized, prospective, double-blind study to investigate the effects of PEMF treatment in 31 patients undergoing knee arthroscopy [70]. Patients were randomized into two groups, control (0.5 mT magnetic field) and active PEMF (1.5 mT magnetic field). The authors did not provide frequency dosimetry data. All patients used the devices for 6 h/day over a 90-day period. KOOS and NSAID use were evaluated before arthroscopy, 45 days postop, and 90 days postop. In addition, 3-year long-term outcome follow-up was conducted through patient interviews. The results demonstrated higher KOOSs in the PEMF group at 45 and 90 days with significant differences at the 90-day period (*p* < 0.05). Significant differences were also found in NSAID use, with 26% of PEMF group patients reporting NSAID use compared to 75% in the control group (*p* = 0.015), indicating a potential decrease in inflammation. At the 3-year follow-up point, there was a significantly greater number of patients in the PEMF group who completely recovered compared to the control group (*p* < 0.05) [70]. The authors concluded that PEMF treatment can improve functional recovery after arthroscopy [70].

Overall, recent research has described the multifaceted role of PEMF in cartilage repair through the regulation of inflammation and promotion of chondrogenesis and ECM synthesis. This review describes PEMF’s treatment ability through the presentation of in vitro and clinical studies, showing the various therapeutic effects of PEMF treatment. The continued translation of preclinical mechanistic studies to long-term human studies can play a crucial role in evolving PEMF as a treatment for OA and cartilage repair.

## 5. Conclusions

PEMFs are in wide use for enhancing bone repair in fracture non-unions, but until relatively recently, the mechanism of membrane signal transduction has been obscure. The observation that PEMFs stimulate adenosine receptors and act as an agonist to the A_2A_ receptor has led to a substantially improved the mechanistic understanding of PEMF signal transduction and also a consideration of the effects of PEMF therapy on tissues and molecules other than in the skeleton. Adenosine receptors are regulators of inflammation and their interactions with PEMF therapy suggest that PEMF therapy may have anti-inflammatory effects that can be exploited clinically. This review is one in a series explaining the mechanisms of action of PEMF therapy on stem cells, endochondral bone formation, and clinical bone repair [71,72,73,74]. This series clarifies the biological effects of PEMF therapy in a number of model systems and should enhance confidence in the mechanistic understanding of PEMF effects clinically. Preclinical and preliminary clinical studies suggest that PEMFs have a spectrum of biological actions including the suppression of inflammation and enhancement of soft tissue repair. What is needed now is a systematic exploration of dosimetry optimization in the several promising tissues and cells responsive to PEMF therapy—in inflammatory joint disease, dermal wound healing, and regenerative medicine.

## Figures and Tables

**Table 1:** Comparison of PEMF vs. sham treatment in diabetic and non-diabetic rats. Data from Goudarzi et al. [17].

Group	Treatment	Wound Healing Time (Days)	Scar Tensile Strength (N/mm^2^)
Diabetic Rats (n = 14)	PEMF (n = 7)	15 *	0.5 **
	Sham (n = 7)	19	0.2
Non-Diabetic Rats (n = 14)	PEMF (n = 7)	13–14 ^+^	0.4 ^++^
	Sham (n = 7)	16–17	0.25

*: Significant reduction in diabetic rat wound healing time; ^+^: Significant reduction in non-diabetic rat wound healing time; **: Significant improvement in diabetic rat scar tensile strength; ^++^: Significant improvement in non-diabetic rat scar tensile strength.

## Data Availability

Not applicable.
